# The virtues in psychiatric treatment

**DOI:** 10.3389/fpsyt.2023.1035530

**Published:** 2023-05-09

**Authors:** John Raymond Peteet

**Affiliations:** Harvard Medical School, Boston, MA, United States

**Keywords:** virtues, ethics, flourishing, positive psychology, professionalism, mental health

## Abstract

Virtues, understood as excellent character traits, originally defined human flourishing, but have been historically neglected within psychiatric practice. Reasons include concerns about scientific objectivity, realistic expectations, and therapeutic moralism. Renewed interest in their clinical relevance has been spurred by problems in sustaining professionalism, growing attention to virtue ethics, empirical support for the benefits of virtues such as gratitude, and the emergence of a fourth wave of growth promoting therapies. Increasing evidence supports the incorporation of a virtues based perspective into diagnostic assessment, goal-setting, and treatment.

## Introduction

Virtues are generally understood to be admirable character traits or stable dispositions to do the right thing for the right reasons, at the right time, in ways that are appropriate to the situation. They involve both thinking and emotion, habit as well as free will, and emerge from a cultural and spiritual context or community which make them intelligible (1). Despite increasing recognition of the value dimension of clinical work, and a growing positive psychology literature on the relationship between character strengths and mental health, the place of virtues in psychiatric treatment has remained relatively unexplored. We review here several historical reasons for this; discuss the ways that virtues are important in diagnostic assessment, goal setting and treatment implementation; and consider the ethical challenges of fostering virtues in treatment.

## Historical background

The term virtue is familiar from its use by the early Greeks, and from Aristotle’s notion of cardinal excellences of character—practical wisdom, self-control, courage, and justice—that are important to living well. Paths toward virtue can also be found in religious writings such as the Bhagavad Gita, Koran, Buddhist, Hebrew and Christian scriptures.

As discussed elsewhere (2), several factors have contributed to the relative neglect of the virtues within psychiatric treatment. Although for centuries physicians were admired for selfless dedication to the care and restoration to wholeness of the sick, Sigmund Freud, influenced by the promise of the Enlightenment, framed the goals of treatment in terms of an objective search for truth and greater individual autonomy, achieved through the exercise of therapeutic neutrality. The technical neutrality of the analyst in evenly hovering over the patient’s conflicts has since often been confused with value neutrality—the notion that the psychotherapist should be value free—which is now generally recognized to be a myth. Furthermore, autonomy understood as freedom from influence has often obscured the more appropriate goal of therapy, autonomy understood as mastery (3). One contributing factor has been what the philosopher Charles Taylor (4) termed the self-sufficient “immanent frame” of a secular age—“an order that requires nothing transcendent in order to function—in which values are defined by rights to be free of constraining influences.” This contrasts with the classical view found in Aristotle and the Judeo-Christian scriptures that freedom depends not on the absence of obstacles, but on the attributes necessary to overcome obstacles and achieve fulfillment—i.e., on character, or virtue.

A second objection to an emphasis on virtues has been Freud’s contention that it is unrealistic: “The commandment, ‘Love thy neighbour as thyself’ is the strongest defence against human aggressiveness and an excellent example of the unpsychological [expectations] of the cultural super-ego. The commandment is impossible to fulfil; such an enormous inflation of love can only lower its value, not get rid of the difficulty” (5). While it is incontestable that humans have the potential for vice as well as virtue, and are not as virtuous as we like to think (6), a viable alternative to Freud’s prescription of lowering standards would seem to be incorporating moral goals into caregiving as appropriate.

This approach was the basis of the Moral Treatment Period in both Europe and then in the U.S. during the 17th and 18th centuries, when reformers such as the founder of the APA Benjamin Rush (7) championed humane ways to encourage rationality and moral strength in asylums. However, as conditions in asylums deteriorated with time, critics such as Foucault argued in Madness and Civilization that the “moral” asylum is “not a free realm of observation, diagnosis, and therapeutics; it is a juridical space where one is accused, judged, and condemned” (8). Foucault was here highlighting a third objection, that an emphasis on virtues can be moralistic, and judgmental. Nevertheless, most clinicians recognize that they cannot avoid moral commitments in their work, and instead of trying to do so should aim to settle on more or less adequate or appropriate ones. Curlin and Hall (9) helpfully point out that moral discourse is often essential to the patient-physician relationship, and that rather than shrinking from such discourse, physicians can best engage patients regarding these concerns not as strangers imposing a technique guided by moral neutrality, but by seeking the patient’s good through “wisdom, candor, and respect.”

A fourth objection raised by materialists since Freud is that virtues, especially inspired by religion, have no place in a scientific discipline such as psychiatry. A number of thinkers (10) and researchers (11) have challenged this assumption, but influential scientists such as Eric Kandel and Joseph Le Doux continue to encourage the reductionistic perspective that the effects of psychotherapy, for example, are best understood in terms of changes in gene expression and neuronal pathways. Perhaps the best response to this reductionistic objection is the relevance of virtues to mental health treatment of the whole person, described below.

## Virtues and mental health

Despite these historical reasons for skepticism regarding the place of virtues within psychiatry, a number of developments have drawn renewed attention to their clinical relevance. First (in part inspired by the fragmented, at times dehumanizing experience of modern medical care), recognition has grown that medicine, including psychiatry, is a value laden enterprise. For example, similar to Values Based Practice, recovery and narrative based approaches, the Dutch philosopher and psychiatrist Gerrit Glas in his recent book Person-centered Care in Psychiatry: Self-relational, Contextual, and Normative Perspectives (12) contends that value-laden assumptions and norms are central in shaping the patient-psychiatrist relationship. A related development is the growth of interest in professionalism, burnout, and moral injury. When clinicians experience moral distress or injury by finding themselves compromised in acquiescing (for example) to productivity demands at the expense of time spent with a patient or family member, they need to cultivate patterns of behavior consistent with their higher values—in other words, character—which in turn reduces future moral injury. Books by Pellegrino and Thomasma (13) and others have described virtues as integral to medical practice, and Radden and Sadler (14) have argued for a virtues based framework as the most appropriate for thinking about psychiatric ethics. Radden and Sadler highlight virtues particularly important for the psychiatrist as including trustworthiness, respect for the patient and for the healing project, empathy and compassion, gender sensitive virtues, warmth, self-knowledge, self-unity, integrity, emotional intelligence, unselfing, realism, authenticity, sincerity, wholeheartedness, and the meta-virtue of phronesis, or practical wisdom.

Second, following on the work of psychologists such as Bergin et al. (15) and Richards et al. (16), calling attention to the place of values in psychotherapy, research psychologists beginning with Fowers (17, 18) have focused on the scientific study of virtue. The field of positive psychology, pioneered by Martin Seligman, now comprises a voluminous literature on the positive mental health outcomes of virtues such as forgiveness, gratitude and hope (19). For example, gratitude interventions are now being shown to improve mental health outcomes through the use of randomized clinical trials, with effects sizes rivaling those of some pharmacological interventions (20). More recently, the virtues of humility (21–23) and practical wisdom (24) have been correlated with improved outcomes in clinical populations. And researchers such as Tyler VanderWeele have launched an emerging science of human flourishing, pointing out that much empirical work in the social and biomedical sciences focuses on narrow outcomes such as a single socioeconomic measure, disease state, or mood state, whereas human well-being or flourishing consists in a much broader range of states and outcomes, including mental and physical health and financial and material security, but also happiness and life satisfaction, meaning and purpose, character and virtue, and close social relationships (25).

Third, such research has helped to inform a fourth wave of therapies that move away from focusing only on psychopathology towards conceptualizing a more positive vision of human flourishing and health (26). This tradition of approaches, built on the legacy of existential psychotherapy and related humanistic approaches, aims beyond insight, mastery and problem solving toward the achievement of positive well being. These increasingly evidence-based value and virtue oriented approaches include positive psychology interventions (PPI), loving kindness and compassion meditation, dignity and gratitude promoting, meaning centered, forgiveness oriented, and spiritually informed therapies. To these could be added Unified Therapy (27, 28), Wong’s meaning therapy (29), Fosha and Thoma’s accelerated experiential dynamic psychotherapy (30), Fava’s well-being therapy (31), and Jeste et al.’s positive psychiatry (32). Furthermore, newer established skills-based therapies, while traditionally considered part of psychology’s Third Wave (33) also foster virtues through their incorporation of mindfulness and acceptance—for example, equanimity in DBT, and courage and practical wisdom in ACT. They overlap with time-honored religious/spiritual (R/S) practices which utilize prayer, scripture study, sacraments, and supportive communities to help individuals achieve valued ideals, and to flourish.

Fourth, contemporary philosophers have helped to clarify the relationship of mental health treatment to education in virtue. Eric Matthews refers to the large number of conditions which lack a clear biological origin, and suggests that in these cases, “‘mental illness’ seems to deviate significantly from the medicalized concept of bodily illness. Deviation from a moral vision of human life seems in these cases to be essential to the definition of what makes a person’s mental condition ‘disordered’: He or she is not simply thinking, feeling, desiring, etc., in ways that differ from what is normal in human beings, but in ways that significantly affect his or her chances of achieving a *satisfactory* human life. …Medical treatment in the form of drugs or even surgery may help to alleviate the effects of such a person’s condition, for example, by making it easier for the person to fit in with conventional society, but the most appropriate treatment for the condition itself …… involves a certain vision of what a satisfactory human life ought to be like, a way of interpreting what is ‘wrong’ with clients as deviation from that ‘moral vision,’ and a way of treating clients aimed at showing them ways of achieving that moral vision in their own lives” (34). The philosopher Duff Waring describes in a more nuanced way a place for therapeutic virtues in psychotherapy understood as the cultivation of character. As Waring (35) (p. 81) puts it, “Virtues are revealed in therapeutic goals that stress the desirable and stable traits of character that mentally healthy persons have and that patients who want to restore lost selves ought to strive for. Hence the idea that mental health amounts to a virtuous state.” He suggests that the realization of certain virtues, e.g., self-love, self-respect, and empathic concern and respect for others, are plausible psychotherapeutic goals for some patients given the problems they present, and that their cultivation and attainment as sufficiently stable states amounts to positive mental health.

The virtues central to Waring’s healing project include perseverance, hopefulness, courage, healing curiosity, and respect for the healing project, as well as focus, and dialogue. He uses the case of a “Demoralized Woman” and an “Angry Man” to illustrate the therapist’s role in cultivating these virtues. “The Demoralized Woman lacks a loving bond with herself by which she knows, feels, and lives by her identity-conferring values and commitments.” In response, the psychotherapist can offer a respectful and alliance in order to reinforce three ideas: “(1) that she is worthy of an ethical identity; (2) that she will discover herself through creative, identity-conferring efforts; and (3) that she can and ought to attend to herself with love and respect.”

Psychodynamic therapy helped The Angry Man who had been abused by his father as a child to recognize the origins of his “simmering antipathy,” but it was when he began to take responsibility for changing it that he experienced the healing virtues of dialogue, empathy and self control. Reminiscent of Matthews, Waring quotes Lomas: “Therapists cannot confront the ‘whole being’ of their patients without asking how they might help that person lead a better life and what that better life might be.” Given his conviction that “there are ways of living that are better than others,” Lomas believed that it was better for his patients not to be crippled by unrealistic fears, consumed by hate, or engaged in persistent lying. He writes that the daily encounters of therapists with patients force them “to enter personal dilemmas of a moral nature,” thus making them “continuously embroiled in questions of morality” and “the kind of life that is worth living” (36). Waring elaborates (p. 98): “This approach to treatment is like a mode of education that requires the active involvement of the patient. At some point, the patient has to accept responsibility for working with, on, and for herself. The working through can involve cultivating affective, cognitive, and behavioral inclinations in the effort to effect morally desirable changes in the way the patient treats other people. It can also involve strenuous efforts at improving oneself by cultivating an appropriate measure of self-love.” Similarly, Radden and Sadler (14) (p. 5) note that virtues such as persistence, courage, honesty, hopefulness, flexibility and trustfulness of the “good patient” are central to the success of treatment. Waring’s list of virtues worth pursuing to achieve treatment goals could be expanded to include those—many now evidence based—of equanimity (mindfulness), forgiveness, gratitude, self-transcendence, defiance, humility, compassion, love, hope (optimism, resilience) and phronesis, or practical wisdom. The effect of therapist characteristics on treatment outcome have begun to receive research attention (37).

### Virtues in treatment

Consider the practical ways that virtues are relevant in psychiatric diagnosis, goal setting and treatment implementation.

#### Diagnostic assessment

A dynamically informed assessment should aim to identify what enduring disposition and/or schema the patient has, and how this constitutes a strength and/or a vulnerability to symptoms and impaired functioning—for example, to caring effectively for him/herself, or regulating emotions well.

Maladaptive personality traits (disorders) are perhaps the most obvious example of a need for virtues—for example, compassion in antisocial personalities, or humility in narcissistic individuals.

A depressive disposition is often marked by guilt, inhibition and impaired self love, reflecting a need for virtues of forgiveness, courage and love.

Anxiety and PTSD are often accompanied by fear and distractibility, reflecting a need for equanimity, courage/defiance, and practical wisdom (phronesis), virtues fostered by approaches such as DBT and ACT.

Substance use disorders are often complicated by impulsivity, irresponsibility and guilt over damage done to self and others, reflecting a need for accountability, gratitude, and self-forgiveness (as encouraged by the Twelve Steps).

Repair of moral injury often entails self awareness, moral integration, and re-connection to community.

#### Goal setting

A comprehensive formulation should not only characterize pathology, but envision a desired, healthier version of the person and consider how the therapist can help them achieve it (a life worth living, in the language of DBT, or in line with one’s personal values, in the language of ACT). Can the depressed patient see a need for hope, or self-forgiveness, or do they need the therapist to lend them hope that a better state of things is possible? Can the anxious patient believe that they can become less obsessional, or do they need to lean on the therapist’s equanimity to begin to trust this possibility? Can the substance using individual accept that a sober, healthy self is worth aiming for, and benefit from the supportive experience of a group promoting virtues such as honesty, accountability and forgiveness? Whether explicitly or by implication, a therapist should develop some picture of the patient’s flourishing toward which to aim, through the acquisition and cultivation of needed virtues.

#### Implementation

In addition to addressing symptoms through medication, insight or support, a clinician should try to identify resources that can be recruited for the patient’s full recovery, resilience and flourishing—both within the therapeutic relationship and from outside it, e.g., in their community, mentors, or experiences of faith. Within the context of treatment, psychodynamic approaches aim at character change through both insight and through corrective emotional experiences with the therapist. Schema therapy explicitly uses the therapeutic relationship to attempts to modify maladaptive ways of being in the world toward more virtuous ones. Cognitive behavioral therapy offers strategies such as practicing healthier perspectives and practices, if need be disputing the patient’s resistances, to achieve change. And fourth wave approaches aimed at growth foster interventions to enhance virtues such as forgiveness or gratitude.

Consider a few ways that an emphasis on virtues can enhance treatment. First, acknowledging the patient’s hopes for living well contextualizes them as a whole person, a task especially pertinent when trauma, adversity or illness focus attention on what matters most (38). Eliciting what the suffering patient most values and hopes to change, including the other-directed aims embodied in most virtues, helps to establish the therapist as a trusted ally in pursuit of not only symptom relief but a more worthwhile life.

*A 37 year old single administrator presented with depression and anxiety of several months duration, concerned that she might never find a partner. Her relationships had followed a pattern of somewhat obsessively pursuing men who showed beginning interest in her, then withdrawing to protect herself from anticipated loss. Having been a practicing Catholic, she now struggled with cynicism and doubt about whether God cared. Exploration traced persisting insecurity to experiences of volatility and harsh criticism from an alcoholic mother. Treatment helped her to see the impact of her childhood experience, and to aim for virtues of equanimity and realistic self-appraisal, drawing on both her supportive father and attentive therapist as models for this. In going further to identify what would be involved in her flourishing, her therapist noted her loss of purpose in her work life, and helped her to explore sources of self-transcendence as a virtue worth recovering. She used this encouragement to engage more fully with colleagues at her mission-based work setting, and in her church.*

Second, transparency about what the therapist values through demonstration of the virtues she embodies can further enhance trust and foster the internalization of qualities important to the patient’s recovery. Radden and Sadler’s explication of the virtues of a good psychiatrist such as trustworthiness, empathy, warmth, self-knowledge, respect, patience and perseverance provide examples of how these inform the therapist’s skillful use of herself (35).

*A divorced woman in her 70s described being depressed and self denigrating for much of her life. She had grown up with a critical mother, and later accommodated the unreasonable demands of an alcoholic husband. Around the time of her divorce, she entered a several year psychotherapy with a psychologist whose consistent interest in her and her experience allowed her to regard herself differently, as worthwhile and capable. In her later years she consolidated this as an enduring stance, giving to her children and others from a sense of generosity rather than obligation.*

Third, it can be helpful to explicitly acknowledge a place for virtues when the patient’s problem has an important moral dimension (38, 39). Examples include a trauma survivor’s struggle with whether to forgive and reconcile; re-evaluation of the individual’s priorities and life direction after a significant loss; disabling shame and guilt with a need of appreciate the virtues one continues to possess; or a disabling lack of a virtue such as accountability (40).

## Discussion

Therapists have differing moral visions, and related preferences for encouraging particular virtues. These may imply differing aims for their patients—e.g., improved functioning, less distress, greater adaptability, enhanced flourishing, greater freedom or deeper relatedness to others. For example, if they believe that flourishing involves pursuit of the Good, understood as what is important beyond the self, relational virtues such as compassion, love and forgiveness will carry more weight than if they envision morality to center on individual rights, and the achievement of autonomy. These differences can be influenced by the therapist’s spiritual tradition. Preferred virtues for Jews often include communal responsibility and critical thought; for Christians, love and grace; for Muslims, reverence and obedience; for Buddhists, equanimity and compassion; for Hindus, appreciation of Dharma and Karma; and for secularists, respect for scientific evidence, autonomy, and intelligibility (41).

The world views and conceptions of the good held by therapists and patients also differ, of course. But this ethical complexity, rather than prompting them to avoid considerations of virtue, should encourage clinicians to (a) be as aware of how their work can be shaped by their moral and spiritual commitments as they are of the potential influence of countertransference, including the risk of imposing their values onto patients; and (b) engage patients regarding these concerns not as strangers imposing a technique guided by moral neutrality, but by discussing and seeking the patient’s good through wisdom, candor, and respect (9).

In summary, a focus on the virtues clarifies how clinical work is both inherently moral and in important ways spiritual, and specifically how treatment that aims to promote human flourishing involves the cultivation of a mature and ethical character. The dynamic relationship between the therapist’s and the patient’s virtues, and how resources outside the treatment can be recruited to cultivate needed virtues are important areas for future study.

## Author contributions

The author confirms being the sole contributor of this work and has approved it for publication.

## Conflict of interest

The author declares that the work was conducted in the absence of any commercial or financial relationships that could be construed as a potential conflict of interest.

## Publisher’s note

All claims expressed in this article are solely those of the authors and do not necessarily represent those of their affiliated organizations, or those of the publisher, the editors and the reviewers. Any product that may be evaluated in this article, or claim that may be made by its manufacturer, is not guaranteed or endorsed by the publisher.
